# A novel computer navigation model guided unilateral percutaneous vertebroplasty for vertebral compression fracture: A case report

**DOI:** 10.1097/MD.0000000000022468

**Published:** 2020-10-30

**Authors:** Hao-Tian Xu, Shuang Zheng, Ming-Yang Kang, Tong Yu, Jian-Wu Zhao

**Affiliations:** Department of Orthopedics, The Second Hospital of Jilin University, Changchun, Jilin Province, China.

**Keywords:** compression fracture, navigation, PKP, screw view model of navigation

## Abstract

**Rational:**

Vertebral compression fracture (VCF) is one of the most common diseases in spinal surgery. Traditional percutaneous vertebroplasty (PVP) under fluoroscopy is an effective method to treat vertebral compression fracture. However, there is still a risk of vascular nerve injury and infection caused by inaccurate or repeated puncture. Therefore, the purpose of this paper was to assess the accuracy of unilateral PVP guided by screw view model of navigation (SVMN) for VCF.

**Patient concerns:**

A 59-year-old female patient suffered high falling injury, and with back pain as its main clinical symptom.

**Diagnoses:**

The patient was diagnosed with a L1 VCF.

**Interventions:**

We placed the puncture needle under the guidance of SVMN to reach the ideal position designed before operation, and then injected the bone cement to complete the percutaneous kyphoplasty (PKP).

**Outcomes:**

The operative time was 29.5 minutes, the puncture time was 1 time, the fluoroscopy time was 2.9 minutes, and the bone cement distribution was satisfactory. VAS and ODI scores were significant improved postoperatively. No surgical complications, including neurovascular injury and infection, were observed during 28-month follow up.

**Lessons:**

The SVMN guided percutaneous puncture needle insertion in PKP operation for VCF is an effective and safety technique. Besides, the SVMN has also been a contributor to reduce radiation doses and replace conventional fluoroscopy.

## 1 Introduction

Vertebral compression fracture (VCF) is one of the most common diseases in spinal surgery, which caused by osteoporosis,^[1]^ high-energy injury^[2]^ and primary or metastatic tumor.^[3,4]^ Traditional percutaneous vertebroplasty (PVP) under fluoroscopy is an effective method to treat vertebral compression fracture.^[5–8]^ However, there is still a risk of vascular nerve injury and infection caused by inaccurate or repeated puncture. Therefore, the purpose of this paper was to assess the accuracy of unilateral PVP guided by screw view model of navigation (SVMN) for VCF.

In recent years, computer navigation was widely used in spinal surgery because it has the characteristics of improving the accuracy of the operation.^[9–12]^ Accordingly, computer navigation image-guided puncture needle for bone cement should be more accurate as well. PKP technology has been skillfully applied by many surgeons. However, authors reported that there are still surgical complications occasionally, including pedicle perforation, asymmetric distribution of bone cement and neurovascular injury caused by inaccurate puncture.^[13,14]^ To improve the accuracy of puncture needle insertion, we applied SVMN in PVP operation, as well we summarized the indications, advantages and disadvantages of this technology.

### 1.1 Ethical approval

This paper was approved by the Second Hospital of Jilin University, Changchun, China. All patients recruited in the paper gave the informed consent.

## 2 Case report

### 2.1 Patient characteristics

**Figure 1:**
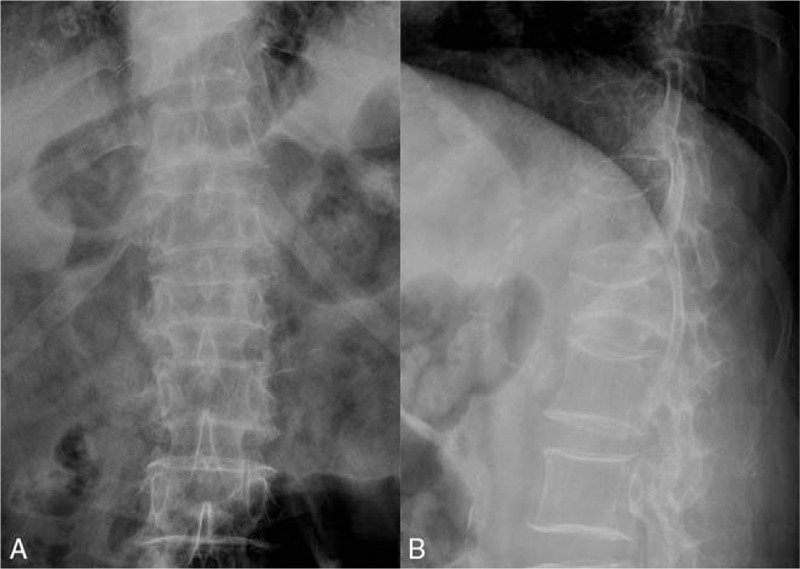
Preoperative X-rays of the lumbar. (A) Anterior-posterior and (B) lateral radiographs showed L1 vertebral compression fractures.

A 59-year-old female patient suffered high falling injury, and with back pain as its predominantly clinical symptom without motor and sensory disorders of the lower extremities (Table 1). Preoperative radiograph films (Fig. 1A-B), computed tomography (CT) and Magnetic resonance imaging (MRI) examination showed L1 fresh VCF. She was diagnosed as L1 VCF.

**Table 1:**
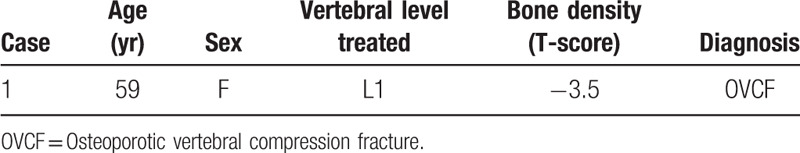
Patient characteristics.

**Figure 2:**
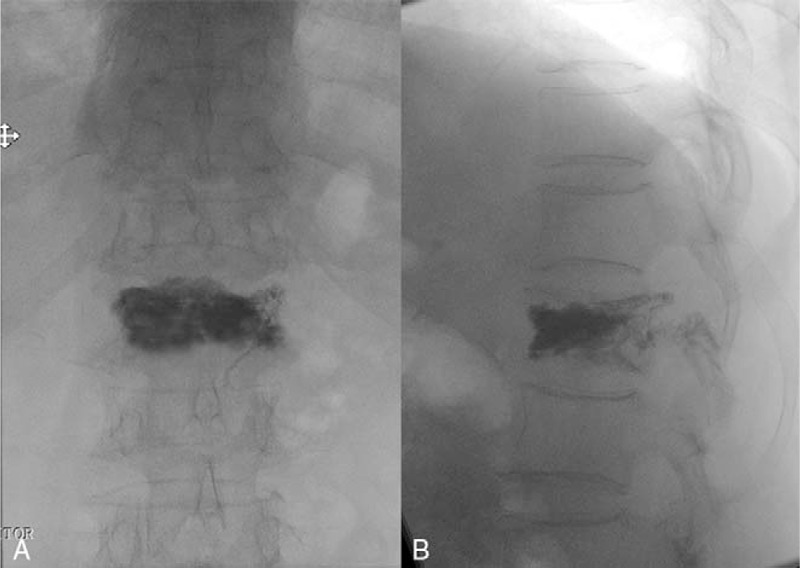
Postoperative X-rays of the lumbar. (A) Frontal and (B) lateral radiographs displayed good dispersion of the bone cement and recovery of the vertebral height.

Operative time, puncture times, fluoroscopy time, the distribution of bone cement was assessed by intraoperative X-rays (Fig. 2A-B) (Table 2). VAS^[15]^ and ODI^[16]^ scores were also evaluated pre- and postoperatively. Moreover, surgical complications were also recorded (Table 3).

**Table 2:**
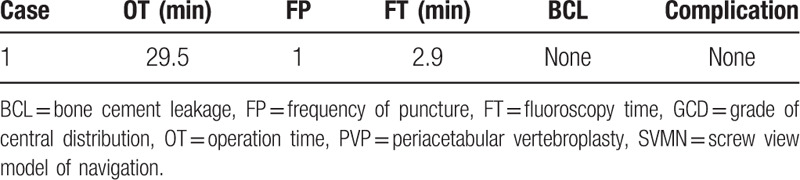
Clinical evaluation indexes of PVP.

**Table 3:**
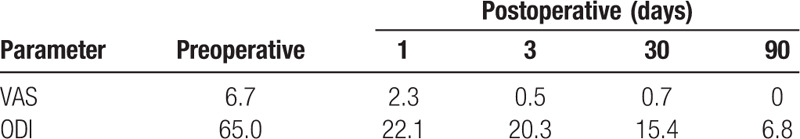
VAS and ODI scores were evacuated preoperative and postoperative.

### 2.2 Surgical technique

#### 2.2.1 Preoperative preparation

**Figure 3:**
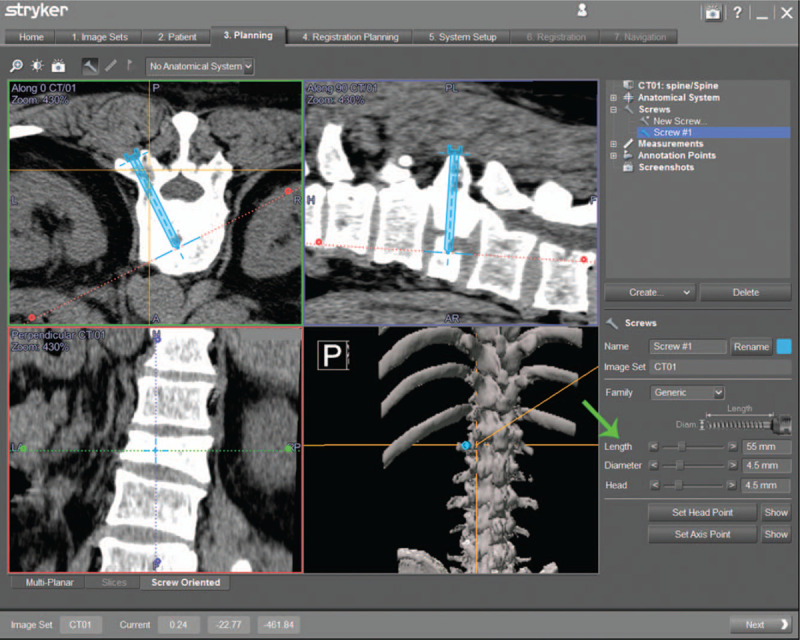
Puncture needle designing. The length and the best trajectory (green arrow) of puncture needle was determined after image acquisition.

The fractured vertebra was scanned by 3D-CT in prone position preoperatively. The image data was recorded in the disc, which could be recognized by a computer navigation system. The entry point of the puncture needle and the best trajectory of the puncture needle were designed at the navigation workstation preoperatively (Fig. 3). The patient was placed in the prone position. To ensure that the relative space between the fracture vertebrae and the operating table remained unchanged, the patient was fixed on the operating table with medical tape by the doctor. Local anesthesia was used with lidocaine (Lidocaine Hydrochloride Injection, 5 ml: 0.1 g, Sui Cheng pharmaceutical Limited by Share Ltd).

#### 2.2.2 Install patient tracker and image acquisition

**Figure 4:**
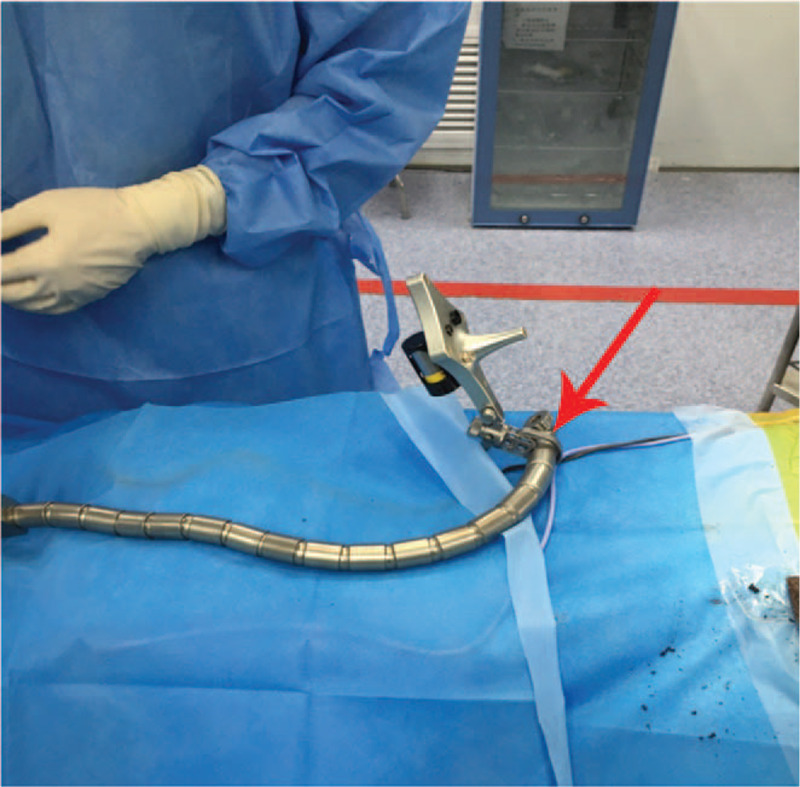
Patient tracker was installed. A patient tracker was fixed on the operating table by the connection of the mechanical arm (red arrow).

**Figure 5:**
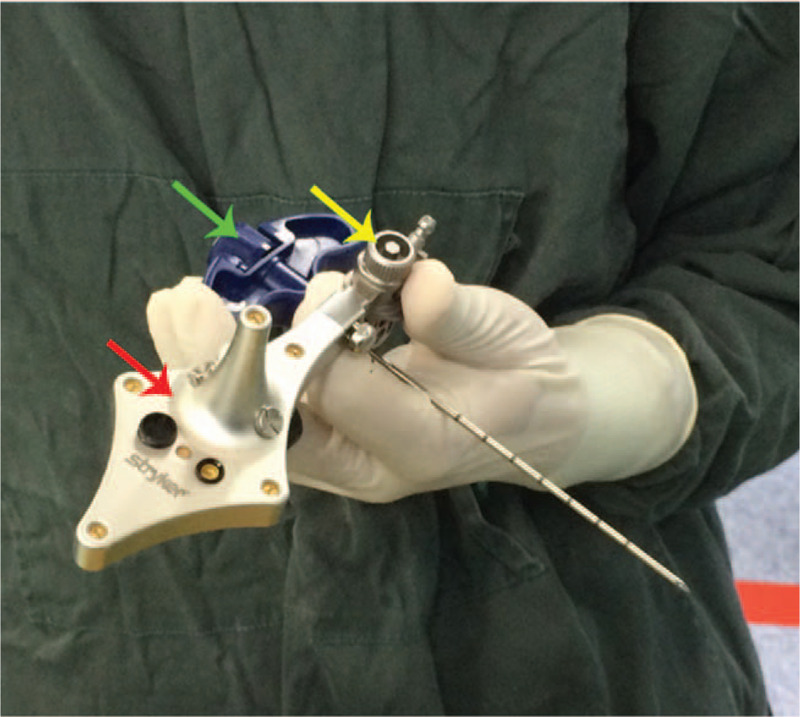
Puncture needle registration in navigation. The instrument tracker (red arrow), indicator (yellow arrow), puncture needle (green arrow), and indicator fixed on the puncture needle and connected to the instrument tracker.

A patient tracker (Stryker Leibinger GmbH & Co., Freiburg, Germany) was fixed on the operating table by the connection of the mechanical arm (Fig. 4). The Navigation System (SpineMap 3D 2.0 software, Stryker Navigation, Kalamazoo, MI, USA) was applied to promote puncture needle insertion. C-arm tracker, patient tracker and puncture needle tracker of the system (Fig. 5) were activated. Fracture vertebra was scanned by C-arm and image information was acquired. To improve the accuracy of puncture needle insertion, we matched the preoperative CT images and intraoperative scanning images to achieve a higher resolution image.

#### 2.2.3 Puncture needle insertion

**Figure 6:**
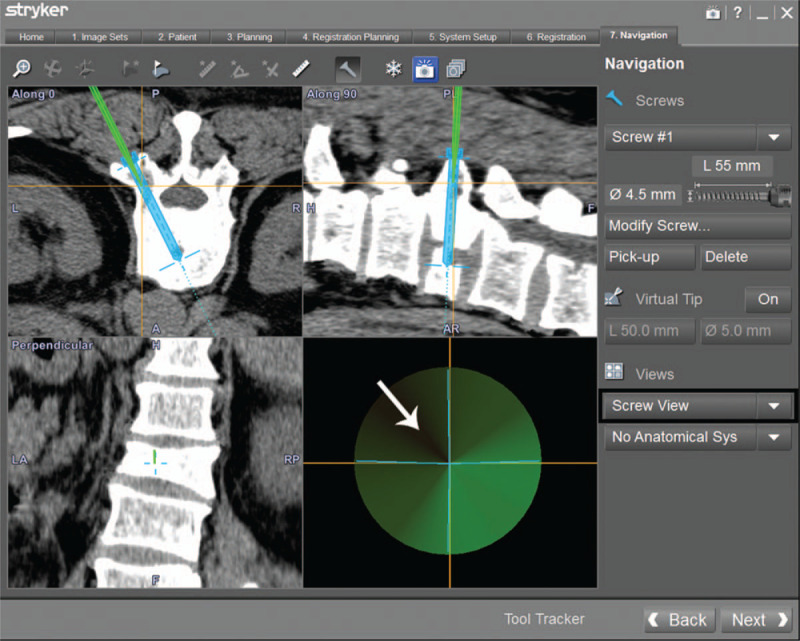
Puncture needle insertion. The screw view model (black border) of navigation was selected on the workstation. It is the most appropriate occasion to implant a puncture needle when the image in right lower shows green (white arrow).

The screw view mode was selected on the navigation workstation. The puncture needle was moved until the direction was completely consistent with the planned position. If the direction of the puncture needle was exactly the same as the preoperative design, the navigation displays green (Fig. 6); In case of slight deviation, the navigation displays yellow; In case of serious deviation, the navigation displays red. The relative spatial position of the patient's tracker and fracture vertebra should not be changed during the operation. After the puncture needle was well positioned, a suitable amount of bone cement was injected.

### 2.3 Results and follow-up

The operative time was 29.5 minutes, the puncture time was 1.09 times, the fluoroscopy time was 2.9 minutes, and the bone cement distribution rate was satisfied. The VAS and ODI were both significantly improved 24 hours after the surgery and during the following up. No surgical complications, including pedicle perforation, cement leakage, neurovascular injury and infection, were observed during 28-month follow up.

## 3 Discussion

Deramond et al^[4]^ in 1984 first described the technique of PVP with polymethylmethacrylate (PMMA) for the treatment of aggressive spinal angiomas. PKP technology has been skillfully applied by many surgeons. However, authors reported that there are still surgical complications occasionally, such as pedicle perforation, cement leakage,^[17–20]^ eccentric distribution of cement and intracardiac bone cement embolism.^[21]^ Among them, most complications are associated with inaccurate insertion of puncture needle. We found that theses clinical complications can be solved by SVMN.

With regard to puncture needle insertion accurately, in the present study, puncture needle trajectory was designed for vertebral compression fractures in computer navigation preoperatively, and the puncture needle was placed accurately under the guidance of SVMN intraoperatively. SVMN shows a perfect puncture needle position on the static axis, sagittal, and coronal image of CT. Moreover, the fracture morphology and navigation of the puncture needle's direction in the small pedicle corridors are clear from the image. As a result, preoperative surgical design and steady intraoperative navigational image were crucial to the whole operation.

For the unilateral or bilateral approach for PVP, currently, authors reported that unilateral PVP has the advantages of short operation time, less radiation exposure and low cost.^[7,22,23]^ However, in unilateral approach PVP, to make the cement distribution across the midline of vertebral body, a larger inclination angle is often needed, increasing the probability of pedicle invasion and nerve injury,^[24]^ even the risk of cement leakage. Consequently, unilateral PVP is the accurate placement of puncture needles. Jianwu et al^[25–30]^ reported SVMN technique can facilitate percutaneous screw implantation in pelvis and spinal surgeries. In this study, puncture needle was inserted under SVMN guidance. There was no significant difference in the improvement of VAS and ODI scores compared with literature,^[6,31,32]^ while the puncture times and the distribution rate of cement were superior to literature.^[8,19,32–34]^ Concerning the radiation exposure, our fluoroscopy time and dose are consistent with the conclusion of Narain et al,^[35]^ which is lower than that of conventional fluoroscopy. Such positive result was attributed to the application of the navigation system for preoperative planning and the guidance of puncture needle insertion with SVMN.

Bone cement leakage is considered a serious complication of PVP,^[8,19,33,34]^ which is likely to cause spinal cord injury^[33,34]^ and even paraplegia. The major factors affecting cement leakage include excessive compression of vertebral body, poor location of puncture needle target, unilateral puncture, repeated insertion of puncture needle, amount of cement injection, and the viscosity of bone cement.^[20]^ In this paper, no bone cement leakage was observed, which was attributed to the accurate placement of puncture needles under SVMN guidance.

Adjacent vertebral body fracture after PVP is a common complication.^[36,37]^ According to the previous literatures, two-thirds of these new fractures occur in vertebrae adjacent to the fractures treated previously.^[38]^ The following factors were considered affecting the occurrence of new fractures: following a vertebral compression fracture, deformity and kyphosis vary the vectors of the forces that are in action throughout the spine.^[39]^ Load-bearing kinetics redistribute the forces to other vertebrae, especially the upper or lower vertebra of the previously fractured vertebra.^[5,39]^ An individual with a normal spine may be able to bear these altered biomechanics, yet a person with a diseased spine may bear these alterations poorly. In this study, no fracture of adjacent vertebral body was found for 28 months follow-up. We attribute this satisfactory result to the early functional reconstruction and calcium supplement to prevent osteoporosis.

Several limitations of this paper are summarized as follows. First, the surgeon should be quite familiar with the state of navigation and judge whether the image is inaccurate to avoid the failure of the operation. Moreover, large number of subjects and further studies need to evaluate the clinical outcomes.

## 4 Conclusion

The SVMN guided percutaneous puncture needle insertion in PKP operation for VCF is an effective and safety technique. Besides, the SVMN has also been a contributor to reduce radiation doses and replace conventional fluoroscopy.

## Acknowledgments

The authors are very grateful to the engineers from the American Stryker company for providing us with the required training skills to use the computer navigation system properly. We also acknowledge the cooperation of the anesthesiologists and nurses in the operating room.

## Author contributions

**Conceptualization:** Jianwu Zhao.

**Data curation:** Tong Yu.

**Formal analysis:** Shuang Zheng, Tong Yu.

**Investigation:** Tong Yu.

**Methodology:** Hao-Tian Xu, Tong Yu.

**Software:** Ming-Yang Kang.

**Supervision:** Jianwu Zhao.

**Writing – original draft:** Hao-Tian Xu, Shuang Zheng, Tong Yu.

**Writing – review & editing:** Jianwu Zhao.
